# The Attitude of Great Writers towards Disease

**Published:** 1892-07-30

**Authors:** 


### THE ATTITUDE OF GREAT WRITERS TOWARDS DISEASE.

### VII.—DANTE.

FEW books have ever received more damage from illustration than has the Inferno, from Doré's weird and wonderful conceptions. And this because many readers to whom Dante is almost unknown even in translations, have shuddered spell-bound over these terrible representations of tortured humanity, and learned from them to believe that the poet revelled in the expression of cruelty, and lowered the strength of his genius to the mere invention of excruciating torments. Whereas in truth Dante found all his horrors ready made to his hand, as Shakespeare did his plots, and needed all his skill to select the least revolting and present them in an artistic form, rather than to pile up ingenious combinations of pain.

There are three main sources from which Dante drew the *mise en scene* of the Inferno. The first is Virgil. From the 6th book of the Æneid is drawn all the classical imagery, Cerberus, Minos, the Furies, the Centaurs, the Minotaur, beings who hold a mysterious second place of interest to the actual *dramatis personæ*. Here, too, is the original of that suburb, if we may call it so, of the City of Dis, where the soft-sighing of infants shut out from bliss, is heard. Another passage of the Æneid suggested the suicides' wood of human trees, and with this is combined the invasion of the Harpies. Here are many elements amplified and spiritualised by Dante. But these are not among the most terrible passages in the Inferno.

Two mediæval writers, at least, before Dante, had attempted a description of the infernal and celestial regions; the monk, Albericus, who was led by St. Peter through the realms of hell and heaven, and the youth, Tantalus, who in the midst of a depraved life was struck down at a banquet and favoured with a vision of the like nature. The date of these writers is uncertain, but they preceded Dante by some centuries, and it is not possible to doubt that he was acquainted with them. Neither of them display much imaginative power. They record a dreary round of tortures on which a savage ingenuity has been exercised. Dante appears to have used these works as a kind of storehouse of horrors, selecting and modifying what he required for the purpose of his allegory. The valley of ice, the stifle of snow mingled with hail, the wells belching out flames, in which those guilty of simony were planted, the great river of hell full of burning pitch, the dark and horrible place full of dragons and serpents, the lake of sulphur guarded by demons, who strike at the souls as they appear above the surface, the gigantic figure of Lucifer, with souls crushed in his hands and squeezed into his mouth, all these features re-appear in the Inferno, but in strangely altered guise, replacing disgust by awe.

The third source open to Dante was that of his own observation. From this is evidently derived his picture of the horrors of war in the ninth compartment of the malebolge, and the punishment of the coiners and forgers by fever, dropsy, and other diseases. He has nothing more terrible than these, because nothing more true to nature.

Dante's observation showed him, indeed, not only that the pains men endure are worse than the pains they set themselves to imagine, but also that every man has judge and executioner ever present with him in the person of his evil deeds, which sentence him even in this life to suffering only confirmed and intensified hereafter. Viewed in this light there is a strong element of retrospect in the Inferno. It is a chapter of real life—a passage back through the wood where for a want of a guide Dante had gone astray, where enlightened by reason (in the person of Virgil), he could now see the despisers of God's law subject to torments not arbitrarily invented, but eternally linked to the crimes they accompany. There is the grandeur of Dante's realm of woe. The chains which bind men to their appointed lot are woven by themselves. The hell is first in their hearts, moulding them insensibly to a condition where the external accidents are a mere reflection of the state to which they have reduced their souls. This view is nowhere more clearly revealed than in the passage where Dante encounters Frate Alberigo and Branca d'Oria in the circle of traitors, knowing them both to be still alive. Both have been guilty of acts of peculiarly cold-blooded treachery, the one by a general assassination in his own house of all his invited guests, the other by the cowardly murder of his daughter's husband. The ice bound condition of the traitors, whose very tears are frozen, fitly symbolizes the pitilessness of their hearts, and their presence there while yet living shows that hell itself could do no more for them than their crimes had already effected.

Thus Dante justifies the ways of God to man, duly recounting the inevitable consequence of unrepented sin, that all (himself not excepted) may receive warning, and that none may be unduly discouraged at the sight of the ungodly in such prosperity. It is all brought terribly near, even to the very thresholds of his hearers. There are his contemporaries of Siena, Padua, Pisa, Bologna, Genoa, above all of Florence (there are twenty-two Florentines) figuring as examples; General, Bishop, Cardinal, Pope, Emperor, all sorts and conditions of men, whose crimes were in every mouth, who had not infrequently exacted respect by the very enormity of their wickedness, and whose lofty station was deemed to justify a special standard of right and wrong. These, till Dante showed them in their true colours, had the world at will. But what miserable creatures they all appear in the Inferno, and not only wretched but mean in their humiliation. How tremendous the force which has succeeded in showing that such they have in fact always been, though tricked out in this life with a few worthless scraps of human greatness.

The effect which Dante intended to produce may be traced by a study of his own attitude in presence of the tortured souls. What kind of pain strikes most dismay in the beholder? Is it not the pain in which he has himself a share? In like manner a punishment which he has run the risk of incurring will fill him with both pity and fear. Now three times in his progress through hell, Dante is overcome with compassion. The first time is when he hears the story of Francesca, and witnesses the torment of the two lovers. The first sight of so many suffering lovers had touched him deeply; he now falls prostrate as one dead. We know that Beatrice accused him of infidelity to her memory, and hold the clue, therefore, to this vivid participation in the lovers' pains. Landor, who seldom looks below the surface in Dante, has a careless little comment on this unusual weakness of Dante's nerves in view of all that lies before him. But his nerves, which carry him steadily through many trying situations, give way once more in presence of the distorted figures of those who have tried to gain unlawful insight into the future. Leaning against a rock he weeps aloud over their shame. We know that he carried his astrological studies far, and that would suffice to render the personal application clear, even if Virgil's reproof were wanting, "Art thou still one of these other fools? Here is pity surviving when it is dead indeed." The third occasion when his tears are stirred is after passing away from those who by civil discord have inflamed the state. Here the tears are not for himself; he pleads in excuse the presence among the throng of one of his own blood, linking him by ties of kinship to association of guilt.

It is this spirit of ready conviction which he would awake in those who accompany him through the dread journey. And joined to this, a certainty that evil in the end never can be triumphant since dominated always by God's great instrument of rectification, which in Dante's philosophy is pain. Pain, linked secretly on earth to men's evil deeds; abiding hereafter with the unrepentant as an instrument of retribution; chastening in the fires of purgatory the redeemed souls as an instrument of purification.

